# *N*′-(3,4-Dichloro­benzyl­idene)-5-methyl-1-(4-nitro­phen­yl)-1*H*-1,2,3-triazole-4-carbohydrazide

**DOI:** 10.1107/S1600536812023112

**Published:** 2012-05-26

**Authors:** Hoong-Kun Fun, Suhana Arshad, Nithinchandra, Balakrishna Kalluraya, J. H. S. Vidyashree

**Affiliations:** a X-ray Crystallography Unit, School of Physics, Universiti Sains Malaysia, 11800 USM, Penang, Malaysia; b Department of Studies in Chemistry, Mangalore University, Mangalagangothri 574 199, Karnataka, India

## Abstract

In the title compound, C_17_H_12_Cl_2_N_6_O_3_, the 1*H*-1,2,3-triazole ring [maximum deviation = 0.003 (1) Å] forms dihedral angles of 34.08 (6) and 28.38 (6)°, respectively, with the nitro- and dichloro-substituted benzene rings. The dihedral angle between the benzene rings is 6.68 (5)°. In the crystal, C—H⋯O hydrogen bonds link the mol­ecules into chains running parallel to the *a* axis.

## Related literature

For aryl hydrazones, see: Sridhar & Perumal (2003 ▶); Bedia *et al.* (2006 ▶); Rollas *et al.* (2002 ▶); Terzioglu & Gürsoy (2003 ▶). For related structures, see: Fun *et al.* (2011 ▶); Wang *et al.* (2010 ▶). For the stability of the temperature controller used in the data collection, see: Cosier & Glazer (1986 ▶).
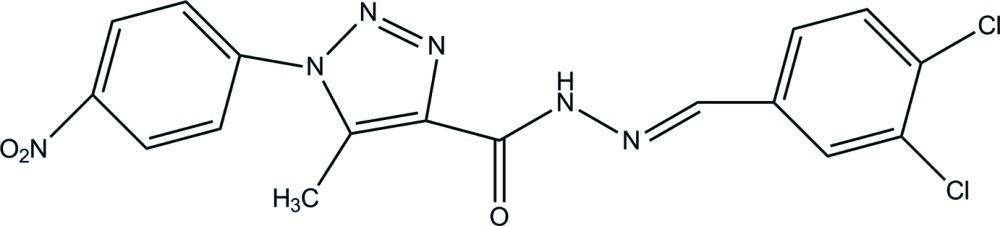


## Experimental

### 

#### Crystal data

C_17_H_12_Cl_2_N_6_O_3_
*M* *_r_* = 419.23
Monoclinic, 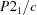

*a* = 6.6309 (3) Å
*b* = 22.7059 (10) Å
*c* = 13.3019 (5) Å
β = 119.559 (2)°
*V* = 1742.08 (13) Å^3^
*Z* = 4
Mo *K*α radiation
μ = 0.41 mm^−1^
*T* = 100 K
0.43 × 0.15 × 0.08 mm

#### Data collection

Bruker SMART APEX DUO CCD diffractometer
Absorption correction: multi-scan (*SADABS*; Bruker, 2009 ▶) *T*_min_ = 0.844, *T*_max_ = 0.967
38004 measured reflections
6085 independent reflections
5280 reflections with *I* > 2σ(*I*)
*R*_int_ = 0.030

#### Refinement

*R*[*F* ^2^ > 2σ(*F* ^2^)] = 0.037
*wR*(*F* ^2^) = 0.099
*S* = 1.04
6085 reflections
258 parameters
H atoms treated by a mixture of independent and constrained refinement
Δρ_max_ = 0.64 e Å^−3^
Δρ_min_ = −0.46 e Å^−3^

### 

Data collection: *APEX2* (Bruker, 2009 ▶); cell refinement: *SAINT* (Bruker, 2009 ▶); data reduction: *SAINT*; program(s) used to solve structure: *SHELXTL* (Sheldrick, 2008 ▶); program(s) used to refine structure: *SHELXTL*; molecular graphics: *SHELXTL*; software used to prepare material for publication: *SHELXTL* and *PLATON* (Spek, 2009 ▶).

## Supplementary Material

Crystal structure: contains datablock(s) global, I. DOI: 10.1107/S1600536812023112/hb6804sup1.cif

Structure factors: contains datablock(s) I. DOI: 10.1107/S1600536812023112/hb6804Isup2.hkl

Supplementary material file. DOI: 10.1107/S1600536812023112/hb6804Isup3.cml

Additional supplementary materials:  crystallographic information; 3D view; checkCIF report

## Figures and Tables

**Table 1:** Hydrogen-bond geometry (Å, °)

*D*—H⋯*A*	*D*—H	H⋯*A*	*D*⋯*A*	*D*—H⋯*A*
C10—H10*A*⋯O3^i^	0.93	2.41	3.2649 (17)	153
C12—H12*A*⋯O3^i^	0.93	2.59	3.4076 (15)	147

Symmetry code: (i) 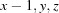.

**Fig. 1.**
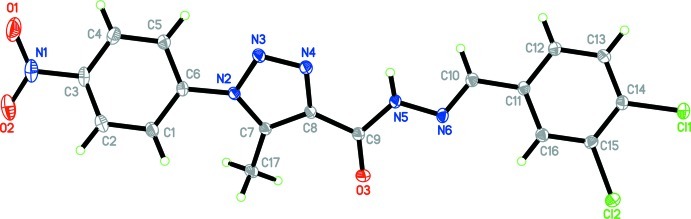
The molecular structure of the title compound, showing 50% probability displacement ellipsoids.

**Fig. 2.**
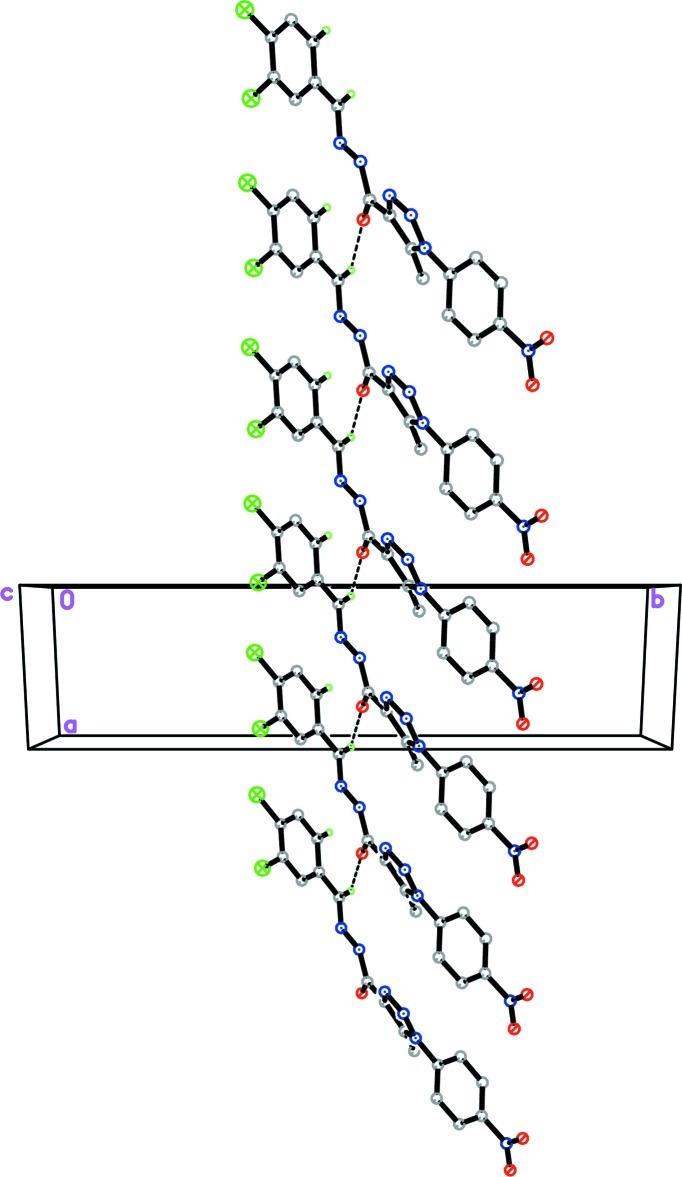
The crystal packing of the title compound, viewed down the c axis. The H atoms not involved in the intermolecular interactions (dashed lines) have been omitted for clarity.

## supplementary crystallographic information

### Comment

Aryl hydrazones are important building blocks for the synthesis of a variety of heterocyclic compounds such as pyrazolines and pyrazoles (Sridhar & Perumal, 2003). Aryl hydrazones have been most conveniently synthesized by the reaction of aryl hydrazines with carbonyl compounds. Hydrazones possessing an azomethine —NHN═CH— proton constitute an important class of compound for new drug development. Hydrazones have been demonstrated to possess anti-microbial, anti-convulsant, analgesic, anti-inflammatory, anti-platelet, anti-tubercular, anti-cancer and anti-tumoral activities (Bedia *et al.*, 2006; Rollas *et al.*, 2002; Terzioglu & Gürsoy, 2003). Prompted by these observations, the title compound was synthesized and its crystal structure is reported here.

The molecular structure is shown in Fig. 1. The 1*H*-1,2,3-triazole ring [N2–N4/C7/C8; maximum deviation of 0.003 (1) Å at atom N3] forms dihedral angles of 34.08 (6) and 28.38 (6)°, respectively with the nitro-substituted and dichloro-substituted phenyl rings (C1–C6 and C11–C16). The dihedral angle between the nitro-substituted (C1–C6) and dichloro-substituted (C11–C16) phenyl rings is 6.68 (5)°. Bond lengths and angles are within normal ranges and comparable to the related structures (Fun *et al.*, 2011; Wang *et al.*, 2010).

The crystal packing is shown in Fig. 2. The molecules are linked *via* intermolecular C10—H10A···O3 and C12—H12A···O3 hydrogen bonds (Table 1) into one-dimensional chain parallel to *a*-axis.

### Experimental

The title compound was obtained by refluxing a mixture of 5-methyl-1- (4-nitrophenyl)-1*H*-1,2,3-triazole-4-carbohydrazide (0.01 mol), 3,4-dichlorobenzaldehyde (0.01 mol) in ethanol (30 ml) and 3 drops of concentrated sulfuric acid for 1 h. Excess ethanol was removed from the reaction mixture under reduced pressure. The solid product obtained was filtered, washed with ethanol and dried. Colourless plates were obtained by slow evaporation of an ethanol-*N*,*N*- dimethylformamide (DMF) (3:1) solution.

### Refinement

The N-bound H atom was located from the difference map and refined freely [N–H = 0.863 (19) Å]. The remaining H atoms were positioned geometrically [C–H = 0.93 or 0.96 Å] and refined using a riding model with *U*_iso_(H) = 1.2 or 1.5 *U*_eq_(C). A rotating group model was applied to the methyl group.

### Crystal data

**Table:**

C_17_H_12_Cl_2_N_6_O_3_	*F*(000) = 856
*M**_r_* = 419.23	*D*_x_ = 1.598 Mg m^−^^3^
Monoclinic, *P*2_1_/*c*	Mo *K*α radiation, λ = 0.71073 Å
Hall symbol: -P 2ybc	Cell parameters from 9957 reflections
*a* = 6.6309 (3) Å	θ = 2.5–32.1°
*b* = 22.7059 (10) Å	µ = 0.41 mm^−^^1^
*c* = 13.3019 (5) Å	*T* = 100 K
β = 119.559 (2)°	Plate, colourless
*V* = 1742.08 (13) Å^3^	0.43 × 0.15 × 0.08 mm
*Z* = 4	

### Data collection

**Table:**

Bruker SMART APEX DUO CCD diffractometer	6085 independent reflections
Radiation source: fine-focus sealed tube	5280 reflections with *I* > 2σ(*I*)
Graphite monochromator	*R*_int_ = 0.030
φ and ω scans	θ_max_ = 32.1°, θ_min_ = 1.8°
Absorption correction: multi-scan (*SADABS*; Bruker, 2009)	*h* = −9→9
*T*_min_ = 0.844, *T*_max_ = 0.967	*k* = −33→33
38004 measured reflections	*l* = −19→19

### Refinement

**Table:**

Refinement on *F*^2^	Primary atom site location: structure-invariant direct methods
Least-squares matrix: full	Secondary atom site location: difference Fourier map
*R*[*F*^2^ > 2σ(*F*^2^)] = 0.037	Hydrogen site location: inferred from neighbouring sites
*wR*(*F*^2^) = 0.099	H atoms treated by a mixture of independent and constrained refinement
*S* = 1.04	*w* = 1/[σ^2^(*F*_o_^2^) + (0.0481*P*)^2^ + 0.9914*P*] where *P* = (*F*_o_^2^ + 2*F*_c_^2^)/3
6085 reflections	(Δ/σ)_max_ = 0.001
258 parameters	Δρ_max_ = 0.64 e Å^−^^3^
0 restraints	Δρ_min_ = −0.46 e Å^−^^3^

### Special details

**Table:**

Experimental. The crystal was placed in the cold stream of an Oxford Cryosystems Cobra open-flow nitrogen cryostat (Cosier & Glazer, 1986) operating at 100.0 (1) K.
Geometry. All e.s.d.'s (except the e.s.d. in the dihedral angle between two l.s. planes) are estimated using the full covariance matrix. The cell e.s.d.'s are taken into account individually in the estimation of e.s.d.'s in distances, angles and torsion angles; correlations between e.s.d.'s in cell parameters are only used when they are defined by crystal symmetry. An approximate (isotropic) treatment of cell e.s.d.'s is used for estimating e.s.d.'s involving l.s. planes.
Refinement. Refinement of *F*^2^ against ALL reflections. The weighted *R*-factor *wR* and goodness of fit *S* are based on *F*^2^, conventional *R*-factors *R* are based on *F*, with *F* set to zero for negative *F*^2^. The threshold expression of *F*^2^ > σ(*F*^2^) is used only for calculating *R*-factors(gt) *etc*. and is not relevant to the choice of reflections for refinement. *R*-factors based on *F*^2^ are statistically about twice as large as those based on *F*, and *R*- factors based on ALL data will be even larger.

### Fractional atomic coordinates and isotropic or equivalent isotropic displacement parameters (Å^2^)

**Table:**

	*x*	*y*	*z*	*U*_iso_*/*U*_eq_	
Cl1	−0.56021 (5)	0.165386 (13)	0.28246 (3)	0.02116 (7)	
Cl2	−0.03571 (5)	0.156467 (14)	0.34211 (3)	0.02223 (8)	
O1	1.5942 (2)	−0.28578 (5)	1.52152 (10)	0.0388 (3)	
O2	1.84421 (19)	−0.26840 (5)	1.46501 (9)	0.0319 (2)	
O3	0.78124 (15)	−0.02047 (4)	0.78857 (7)	0.01839 (17)	
N1	1.6545 (2)	−0.26107 (5)	1.45880 (10)	0.0250 (2)	
N2	1.01583 (16)	−0.11155 (4)	1.10939 (8)	0.01214 (16)	
N3	0.83699 (17)	−0.09041 (4)	1.12221 (8)	0.01438 (17)	
N4	0.70655 (17)	−0.05895 (4)	1.03092 (8)	0.01456 (17)	
N5	0.45947 (17)	−0.01568 (4)	0.81069 (8)	0.01497 (18)	
N6	0.32662 (17)	0.01542 (4)	0.71002 (8)	0.01402 (17)	
C1	1.4138 (2)	−0.14706 (5)	1.22695 (10)	0.0166 (2)	
H1A	1.4642	−0.1214	1.1894	0.020*	
C2	1.5691 (2)	−0.18447 (5)	1.31329 (10)	0.0188 (2)	
H2A	1.7244	−0.1852	1.3327	0.023*	
C3	1.4894 (2)	−0.22062 (5)	1.36985 (10)	0.0183 (2)	
C4	1.2597 (2)	−0.22145 (5)	1.34402 (10)	0.0196 (2)	
H4A	1.2119	−0.2453	1.3851	0.024*	
C5	1.1028 (2)	−0.18563 (5)	1.25507 (10)	0.0165 (2)	
H5A	0.9466	−0.1862	1.2339	0.020*	
C6	1.18123 (19)	−0.14880 (5)	1.19790 (9)	0.01316 (19)	
C7	0.99791 (19)	−0.09356 (5)	1.00752 (9)	0.01230 (18)	
C8	0.79878 (19)	−0.05976 (5)	0.95886 (9)	0.01275 (18)	
C9	0.68418 (19)	−0.02983 (5)	0.84506 (9)	0.01328 (19)	
C10	0.1116 (2)	0.01900 (5)	0.68224 (9)	0.01384 (19)	
H10A	0.0589	−0.0010	0.7258	0.017*	
C11	−0.05113 (19)	0.05422 (5)	0.58286 (9)	0.01318 (18)	
C12	−0.2838 (2)	0.05721 (5)	0.55521 (10)	0.0160 (2)	
H12A	−0.3340	0.0366	0.5993	0.019*	
C13	−0.4402 (2)	0.09107 (5)	0.46155 (10)	0.0179 (2)	
H13A	−0.5953	0.0925	0.4423	0.021*	
C14	−0.3645 (2)	0.12262 (5)	0.39687 (10)	0.0152 (2)	
C15	−0.1323 (2)	0.11926 (5)	0.42388 (9)	0.01465 (19)	
C16	0.0234 (2)	0.08516 (5)	0.51611 (9)	0.01451 (19)	
H16A	0.1774	0.0828	0.5336	0.017*	
C17	1.1550 (2)	−0.11109 (6)	0.96271 (10)	0.0177 (2)	
H17A	1.0738	−0.1081	0.8799	0.027*	
H17B	1.2053	−0.1510	0.9850	0.027*	
H17C	1.2874	−0.0855	0.9942	0.027*	
H1N5	0.406 (3)	−0.0207 (8)	0.8574 (16)	0.027 (5)*	

### Atomic displacement parameters (Å^2^)

**Table:**

	*U*^11^	*U*^22^	*U*^33^	*U*^12^	*U*^13^	*U*^23^
Cl1	0.01639 (13)	0.02378 (14)	0.02034 (14)	0.00532 (10)	0.00678 (11)	0.00923 (10)
Cl2	0.02019 (14)	0.02874 (15)	0.02075 (14)	0.00390 (11)	0.01239 (12)	0.01002 (10)
O1	0.0315 (6)	0.0358 (6)	0.0322 (6)	0.0003 (5)	0.0026 (5)	0.0195 (5)
O2	0.0246 (5)	0.0302 (5)	0.0272 (5)	0.0129 (4)	0.0023 (4)	0.0007 (4)
O3	0.0149 (4)	0.0252 (4)	0.0170 (4)	0.0007 (3)	0.0093 (3)	0.0048 (3)
N1	0.0232 (5)	0.0183 (5)	0.0197 (5)	0.0029 (4)	−0.0001 (4)	0.0014 (4)
N2	0.0103 (4)	0.0142 (4)	0.0116 (4)	0.0010 (3)	0.0051 (3)	0.0001 (3)
N3	0.0127 (4)	0.0173 (4)	0.0140 (4)	0.0033 (3)	0.0072 (3)	0.0011 (3)
N4	0.0131 (4)	0.0179 (4)	0.0131 (4)	0.0028 (3)	0.0067 (3)	0.0019 (3)
N5	0.0131 (4)	0.0208 (4)	0.0111 (4)	0.0039 (3)	0.0061 (3)	0.0044 (3)
N6	0.0137 (4)	0.0161 (4)	0.0103 (4)	0.0028 (3)	0.0045 (3)	0.0014 (3)
C1	0.0128 (5)	0.0183 (5)	0.0160 (5)	0.0002 (4)	0.0049 (4)	0.0005 (4)
C2	0.0126 (5)	0.0202 (5)	0.0182 (5)	0.0026 (4)	0.0035 (4)	−0.0007 (4)
C3	0.0175 (5)	0.0150 (5)	0.0135 (5)	0.0033 (4)	0.0007 (4)	0.0005 (4)
C4	0.0206 (6)	0.0176 (5)	0.0157 (5)	−0.0011 (4)	0.0052 (4)	0.0031 (4)
C5	0.0134 (5)	0.0186 (5)	0.0149 (5)	−0.0007 (4)	0.0051 (4)	0.0016 (4)
C6	0.0124 (5)	0.0128 (4)	0.0113 (4)	0.0010 (3)	0.0035 (4)	−0.0004 (3)
C7	0.0106 (4)	0.0142 (4)	0.0111 (4)	−0.0003 (3)	0.0047 (4)	−0.0001 (3)
C8	0.0105 (4)	0.0152 (4)	0.0122 (4)	0.0009 (3)	0.0053 (4)	0.0006 (3)
C9	0.0118 (5)	0.0149 (4)	0.0121 (4)	0.0004 (4)	0.0051 (4)	0.0005 (3)
C10	0.0143 (5)	0.0147 (4)	0.0120 (4)	0.0010 (4)	0.0062 (4)	0.0001 (3)
C11	0.0127 (5)	0.0142 (4)	0.0112 (4)	0.0010 (4)	0.0048 (4)	−0.0003 (3)
C12	0.0134 (5)	0.0183 (5)	0.0153 (5)	0.0006 (4)	0.0062 (4)	0.0029 (4)
C13	0.0121 (5)	0.0217 (5)	0.0189 (5)	0.0016 (4)	0.0070 (4)	0.0046 (4)
C14	0.0141 (5)	0.0159 (4)	0.0140 (4)	0.0020 (4)	0.0057 (4)	0.0020 (4)
C15	0.0162 (5)	0.0153 (4)	0.0136 (4)	0.0005 (4)	0.0083 (4)	0.0012 (3)
C16	0.0137 (5)	0.0167 (4)	0.0133 (4)	0.0013 (4)	0.0067 (4)	0.0003 (3)
C17	0.0139 (5)	0.0250 (5)	0.0169 (5)	0.0030 (4)	0.0096 (4)	0.0012 (4)

### Geometric parameters (Å, º)

**Table:**

Cl1—C14	1.7305 (11)	C4—C5	1.3889 (16)
Cl2—C15	1.7304 (11)	C4—H4A	0.9300
O1—N1	1.2248 (17)	C5—C6	1.3920 (16)
O2—N1	1.2303 (17)	C5—H5A	0.9300
O3—C9	1.2252 (14)	C7—C8	1.3813 (15)
N1—C3	1.4711 (15)	C7—C17	1.4874 (16)
N2—C7	1.3631 (14)	C8—C9	1.4818 (15)
N2—N3	1.3658 (13)	C10—C11	1.4642 (15)
N2—C6	1.4249 (14)	C10—H10A	0.9300
N3—N4	1.3024 (13)	C11—C16	1.3998 (15)
N4—C8	1.3681 (14)	C11—C12	1.4000 (16)
N5—C9	1.3640 (14)	C12—C13	1.3941 (16)
N5—N6	1.3799 (13)	C12—H12A	0.9300
N5—H1N5	0.863 (19)	C13—C14	1.3897 (16)
N6—C10	1.2871 (15)	C13—H13A	0.9300
C1—C2	1.3906 (16)	C14—C15	1.4001 (16)
C1—C6	1.3935 (16)	C15—C16	1.3858 (15)
C1—H1A	0.9300	C16—H16A	0.9300
C2—C3	1.3824 (18)	C17—H17A	0.9600
C2—H2A	0.9300	C17—H17B	0.9600
C3—C4	1.3869 (18)	C17—H17C	0.9600
			
O1—N1—O2	123.98 (12)	N4—C8—C7	109.48 (9)
O1—N1—C3	118.05 (12)	N4—C8—C9	121.89 (10)
O2—N1—C3	117.97 (12)	C7—C8—C9	128.57 (10)
C7—N2—N3	111.32 (9)	O3—C9—N5	124.82 (10)
C7—N2—C6	130.79 (9)	O3—C9—C8	123.11 (10)
N3—N2—C6	117.85 (9)	N5—C9—C8	112.05 (9)
N4—N3—N2	107.12 (9)	N6—C10—C11	120.81 (10)
N3—N4—C8	108.97 (9)	N6—C10—H10A	119.6
C9—N5—N6	120.97 (9)	C11—C10—H10A	119.6
C9—N5—H1N5	120.0 (12)	C16—C11—C12	119.81 (10)
N6—N5—H1N5	118.2 (12)	C16—C11—C10	120.89 (10)
C10—N6—N5	113.41 (10)	C12—C11—C10	119.30 (10)
C2—C1—C6	118.62 (11)	C13—C12—C11	119.94 (11)
C2—C1—H1A	120.7	C13—C12—H12A	120.0
C6—C1—H1A	120.7	C11—C12—H12A	120.0
C3—C2—C1	119.09 (11)	C14—C13—C12	120.06 (11)
C3—C2—H2A	120.5	C14—C13—H13A	120.0
C1—C2—H2A	120.5	C12—C13—H13A	120.0
C2—C3—C4	122.77 (11)	C13—C14—C15	120.02 (10)
C2—C3—N1	118.42 (11)	C13—C14—Cl1	119.38 (9)
C4—C3—N1	118.78 (11)	C15—C14—Cl1	120.60 (9)
C3—C4—C5	118.19 (11)	C16—C15—C14	120.16 (10)
C3—C4—H4A	120.9	C16—C15—Cl2	118.96 (9)
C5—C4—H4A	120.9	C14—C15—Cl2	120.88 (8)
C4—C5—C6	119.53 (11)	C15—C16—C11	120.00 (10)
C4—C5—H5A	120.2	C15—C16—H16A	120.0
C6—C5—H5A	120.2	C11—C16—H16A	120.0
C5—C6—C1	121.73 (10)	C7—C17—H17A	109.5
C5—C6—N2	117.74 (10)	C7—C17—H17B	109.5
C1—C6—N2	120.52 (10)	H17A—C17—H17B	109.5
N2—C7—C8	103.11 (9)	C7—C17—H17C	109.5
N2—C7—C17	125.69 (10)	H17A—C17—H17C	109.5
C8—C7—C17	131.11 (10)	H17B—C17—H17C	109.5
			
C7—N2—N3—N4	−0.47 (12)	N3—N4—C8—C9	177.51 (10)
C6—N2—N3—N4	−178.56 (9)	N2—C7—C8—N4	−0.28 (12)
N2—N3—N4—C8	0.28 (12)	C17—C7—C8—N4	176.31 (11)
C9—N5—N6—C10	171.91 (10)	N2—C7—C8—C9	−177.56 (10)
C6—C1—C2—C3	−2.09 (17)	C17—C7—C8—C9	−1.0 (2)
C1—C2—C3—C4	0.21 (18)	N6—N5—C9—O3	−5.22 (17)
C1—C2—C3—N1	178.39 (11)	N6—N5—C9—C8	176.32 (9)
O1—N1—C3—C2	168.22 (12)	N4—C8—C9—O3	166.12 (11)
O2—N1—C3—C2	−12.38 (17)	C7—C8—C9—O3	−16.90 (18)
O1—N1—C3—C4	−13.52 (18)	N4—C8—C9—N5	−15.39 (15)
O2—N1—C3—C4	165.87 (12)	C7—C8—C9—N5	161.59 (11)
C2—C3—C4—C5	2.01 (18)	N5—N6—C10—C11	175.27 (9)
N1—C3—C4—C5	−176.16 (11)	N6—C10—C11—C16	−0.42 (16)
C3—C4—C5—C6	−2.29 (18)	N6—C10—C11—C12	−179.89 (10)
C4—C5—C6—C1	0.43 (17)	C16—C11—C12—C13	−0.14 (17)
C4—C5—C6—N2	−178.26 (10)	C10—C11—C12—C13	179.34 (10)
C2—C1—C6—C5	1.80 (17)	C11—C12—C13—C14	−1.09 (18)
C2—C1—C6—N2	−179.55 (10)	C12—C13—C14—C15	1.62 (18)
C7—N2—C6—C5	−144.57 (12)	C12—C13—C14—Cl1	−178.90 (9)
N3—N2—C6—C5	33.08 (14)	C13—C14—C15—C16	−0.91 (17)
C7—N2—C6—C1	36.73 (17)	Cl1—C14—C15—C16	179.61 (9)
N3—N2—C6—C1	−145.62 (11)	C13—C14—C15—Cl2	178.15 (9)
N3—N2—C7—C8	0.46 (12)	Cl1—C14—C15—Cl2	−1.32 (14)
C6—N2—C7—C8	178.23 (10)	C14—C15—C16—C11	−0.32 (17)
N3—N2—C7—C17	−176.38 (10)	Cl2—C15—C16—C11	−179.41 (8)
C6—N2—C7—C17	1.40 (18)	C12—C11—C16—C15	0.84 (16)
N3—N4—C8—C7	0.01 (13)	C10—C11—C16—C15	−178.62 (10)

### Hydrogen-bond geometry (Å, º)

**Table:**

*D*—H···*A*	*D*—H	H···*A*	*D*···*A*	*D*—H···*A*
C10—H10*A*···O3^i^	0.93	2.41	3.2649 (17)	153
C12—H12*A*···O3^i^	0.93	2.59	3.4076 (15)	147

Symmetry code: (i) *x*−1, *y*, *z*.
